# Impact of Bariatric Surgical Intervention on Peripheral Blood Neutrophil (PBN) Function in Obesity

**DOI:** 10.1007/s11695-017-3063-1

**Published:** 2017-12-14

**Authors:** Helen M. Roberts, Melissa M. Grant, Naomi Hubber, Paul Super, Rishi Singhal, Iain L. C. Chapple

**Affiliations:** 1 Periodontal Research Group, School of Dentistry, Institute of Clinical Science, University of Birmingham and Birmingham Dental Hospital (Birmingham Community Healthcare Trust), 5 Mill Pool Way, Edgbaston, Birmingham, B5 7ET UK; 2 Birmingham Heartlands Hospital, Bordesley Green East, B9 5SS Birmingham, UK

**Keywords:** Chemotaxis, Glycated haemoglobin (HbA1c), Neutrophil extracellular traps (NETs), Peripheral blood neutrophil (PBN), Reactive oxygen species (ROS), Very low-calorie diet (VLCD), Cytokines

## Abstract

**Aim:**

The aim of this study was to investigate the impact of weight loss following gastric band surgery on multiple measures of peripheral blood neutrophil (PBN) function.

**Material and Methods:**

Twenty-three obese patients undergoing gastric band surgery were recruited to a longitudinal intervention study, alongside non-obese, healthy gender- and age-matched controls. Eighteen pairs of patients and controls completed all stages of the study. PBNs were isolated by density centrifugation and a comprehensive analysis of PBN function was undertaken at various stages of the patients’ bariatric surgical care pathway.

**Results:**

Obese patients exhibited exaggerated PBN activity in response to various stimuli, characterised by higher reactive oxygen species (ROS) generation (*n* = 18, *p* < 0.001) and release of pro-inflammatory cytokines (*n* = 10, *p* < 0.05) and lower PBN extracellular trap (NET) formation (*n* = 18, *p* < 0.01). PBN chemotactic accuracy was also impaired prior to surgery (*n* = 18, *p* < 0.01). Weight loss was associated with normalised NET production and lower ROS production and cytokine release relative to healthy controls. However, chemotactic accuracy remained impaired in patients.

**Conclusions:**

Weight loss following gastric band surgery was associated with a decrease in the pro-inflammatory activities of peripheral blood neutrophils (PBNs). A hyper-inflammatory PBN phenotype, involving excess ROS and cytokine release, reduced NET formation and chemotaxis, may lead to a reduced ability to eliminate infection, alongside inflammation-mediated tissue damage in obese individuals.

**Electronic supplementary material:**

The online version of this article (10.1007/s11695-017-3063-1) contains supplementary material, which is available to authorized users.

## Introduction

Worldwide obesity rates have almost doubled since 1980 with the highest prevalence rates being in developed countries including the USA and Europe [1]. 3.4 million adults per year die from obesity-related complications [2], and obesity is strongly associated with a range of co-morbid conditions including type 2 diabetes, hypercholesterolaemia, hypertension and obstructive sleep apnoea [3]. Obesity has been shown to associate with increased mortality rates in influenza [4], the incidence of urinary tract infections [5] and nosocomial infections [6], and has positive associations with periodontitis prevalence [7]. Possible underlying mechanisms of association include immune system dysregulation, reduced cell-mediated immune responses, obesity-related co-morbidities and altered pharmacological responses [8]. Bariatric surgery is now considered as a first-line treatment for weight loss in individuals with a BMI of ≥ 35 who have co-morbidities and a BMI of > 40 in the absence of co-morbidity [9].

Peripheral blood neutrophils (PBNs) are sentinels of innate immune responses to infectious agents and the impact of obesity upon PBN function is therefore worthy of characterisation. PBNs infiltrate inflamed/infected tissues in increasing numbers in response to a diverse range of immunogens and are recruited from the circulation by a series of chemoattractants, released initially from the inflamed tissues, which induce gradient-driven chemotaxis. Upon pathogenic challenge, PBNs are equipped with a variety of killing mechanisms, including the release of reactive oxygen species (ROS) [10], pro-inflammatory cytokines [11] and neutrophil extracellular traps (NETs), which serve to trap bacteria, facilitating their destruction [12]. Whilst integral to an effective immune response, dysregulated PBN behaviour has been reported in many chronic inflammatory conditions including rheumatoid arthritis [13], diabetes [14] and certain cancers [15].

Therefore, this study aimed to comprehensively analyse PBN function in morbidly obese individuals prior to and following gastric band surgery (laparoscopic adjustable gastric band), alongside lean gender- and age-matched healthy controls.

## Materials and Methods

### Study Population

Ethical approval for the study was obtained from the North West Research Ethics Committee (15/NW/07). Patient and control exclusion criteria included pregnancy and smoking. Gender- and age-matched healthy controls were recruited from staff within the School of Dentistry, University of Birmingham. Exclusion criteria for controls included a BMI over 27, pregnancy, smoking, evidence of systemic disease and use of medications/vitamin supplements. Informed consent was obtained from all individual participants included in the study.

### Clinical Pathway for Patients

Patients were initially referred to the medical weight management services at the Birmingham Heartlands Hospital by their general medical practitioner. After spending a defined time period in the weight management clinics (12–18 months), patients were referred for bariatric surgery. Patients were offered a choice of three bariatric procedures (laparoscopic adjustable gastric banding, laparoscopic sleeve gastrectomy and laparoscopic Roux-en-Y gastric bypass). To avoid outcome confounding arising due to the different surgical approaches, only patients scheduled for gastric banding were offered recruitment into the current study. All patients were required to engage with a very low-calorie diet (VLCD) for a period of approximately 8 weeks prior to the procedure (time point 1, T1). The VLCD is part of the treatment regimen as previous studies have demonstrated reduced post-operative complication rates in patients [16, 17]. This was typically 600–800 kcal/day for that defined period. Patients were usually pre-assessed for surgery 2 weeks prior to the operation date (time point 2, T2). Patients were admitted on the day of surgery and following discharge followed up in the dietetic clinics approximately 3 months later (time point 3, T3).

**Fig. 1:**
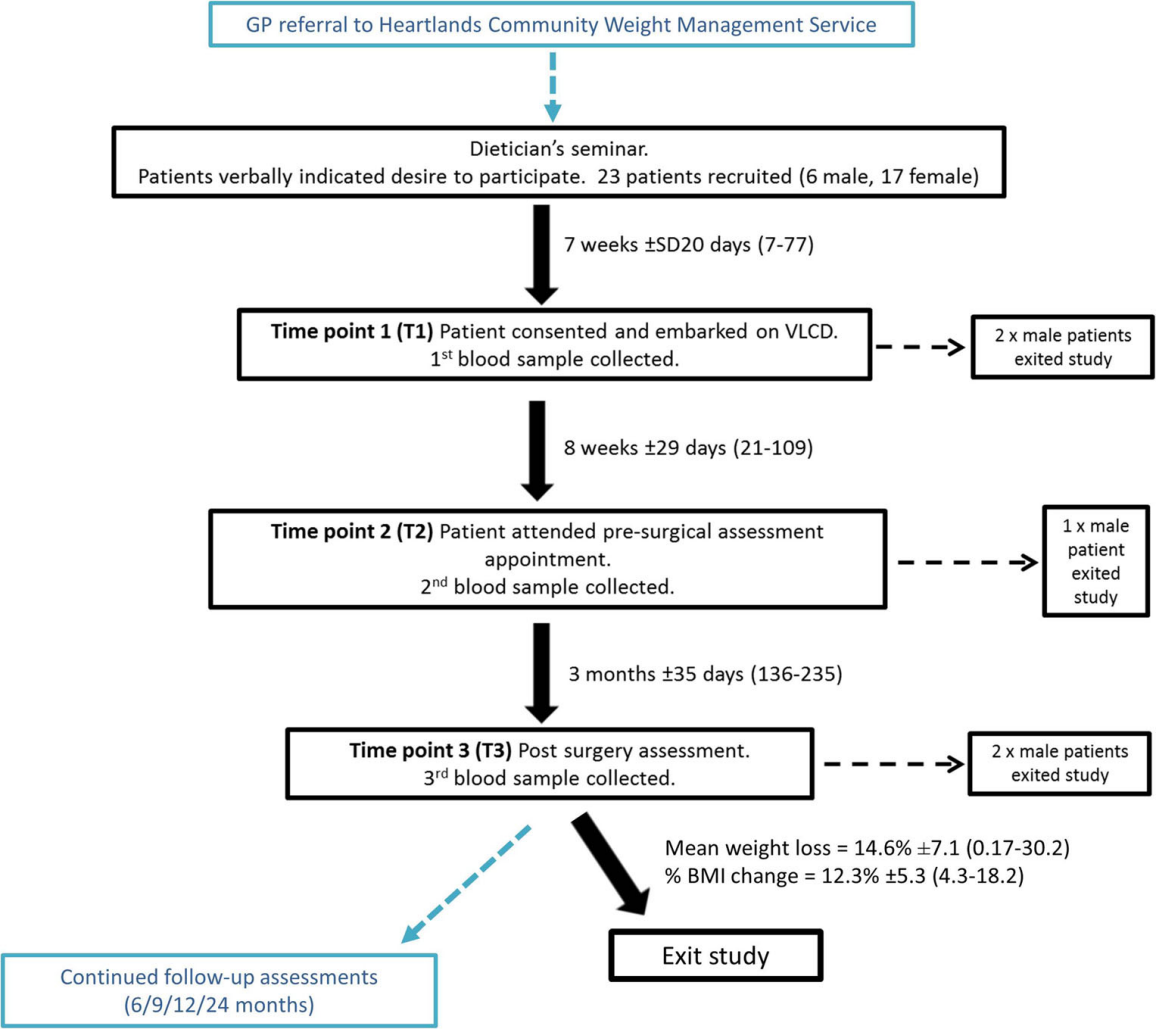
Participant flow diagram. Patients were recruited within the bariatric surgical pathway; all appointments that coincided with study involvement are highlighted in black. Obese participants were initially recruited verbally during the dietician’s session that laid out treatment options. Participants were required to follow a very low-calorie diet (VLCD) of their choice (Cambridge Weight Plan®, Weightwatchers® or restriction to 1000 cal per day by consuming a varied diet or a diet limited to foods, such as milk and yoghurt). Blood samples were taken at three different time points coinciding with patient attendance at bariatric clinical appointments. Time/weight changes shown as mean, ±standard deviation and range

Figure 1 summarises the pathway through the study, which involved three blood donations at time points within the treatment timeline: prior to a VLCD (T1), pre-surgery (T2) and post-surgery (T3). BMI (kg/m^2^) was calculated at each study visit for each participant.

### Collection of Blood and PBN Preparation

Venous blood (24 ml) was collected from the ante-cubital fossa into Vacutainer™ lithium heparin (17 IU/ml) tubes, and PBNs were isolated using Percoll density gradients (GE Healthcare) as previously described [18]. Cell viability, typically > 98%, was determined by dye exclusion (trypan blue). Cell purity was determined by cytospin and fluorescence-activated cell sorting (FACS) on the basis of forward- and side-scatter characteristics [19].

### Blood Chemistry Measurements and Plasma Preparation

Glycated haemoglobin (HbA1c) was measured using a HemoCue® HbA1c 501 blood analyser (HemoCue, Sweden) from whole blood at all three time points. Blood lipids high-density lipoprotein (HDL), low-density lipoprotein (LDL), cholesterol and triglycerides (TG) were measured in whole blood using a CardioChek PA Blood Analyser (BHR Pharma, UK). Plasma was isolated from blood by centrifugation (30 min, 1000 rcf, 4 °C; IEC Centra, CL3R), aliquoted (500 μl) into cryotubes and stored at − 80 °C.

### Bacterial Stimulus

Bacterial stimuli employed for the PBN assays included the oral bacterial species, *Streptococcus constellatus* (*S. constellatus*; ATCC 43146), *Fusobacterium nucleatum* (*F. nucleatum*; ATCC 10953) and *Staphylococcus aureus* (*S. aureus*; ATCC 9144) for toll-like receptors. *S. aureus* was opsonised as previously described [20] to facilitate Fcγ-receptor stimulation.

### Neutrophil Extracellular Trap Release

Neutrophil Extracellular Traps (NETs) were quantified as described previously [21]. Briefly, isolated PBNs were stimulated with *S. constellatus* (MOI of 300:1), phorbol myristate acetate (PMA; 50 nM) or hypochlorous acid (HOCl) (0.75 mM). Following a 3-h incubation, released NET-DNA was digested with micrococcal nuclease and NETs were detected using Sytox green (Life Technologies; 10 μM). Fluorescence was measured in arbitrary fluorescence units (AFU) using a Twinkle LB970 fluorimeter (Berthold Technologies, USA).

### Reactive Oxygen Species Production

Reactive oxygen species (ROS) production was measured using luminol, isoluminol and lucigenin as substrates for detecting total HOCl, extracellular HOCl and extracellular superoxide anion production, respectively, as previously described [20]. Briefly, PBNs were primed for 30 min with either GM-CSF (10 ng/ml, equivalent to 100 pg/10^5^ cells) or PBS and then stimulated with *F. nucleatum* (MOI 300:1) or opsonised *Staphyloccocus aureus* (MOI of 150:1) for 120 min. Peak RLU values were determined pre- and post-priming/stimulation using a TriStar luminometer (Berthold Technologies, USA).

### PBN Chemotaxis

Chemotaxis was measured by real-time video microscopy using the Insall bridge chamber as previously described [22]. Briefly, isolated PBNs were introduced into an Insall chamber and cell movement towards the chemoattractants N-formylmethionyl-leucyl-phenylalanine (fMLP, 10 nM) and interleukin 8 (CXCL8, 200 ng/ml) analysed using a Zeiss Primovert microscope (Carl Zeiss Imaging, Thornwood, NY, USA). The resultant numerical data generated were used to measure chemotactic accuracy, defined in terms of speed, velocity (directed movement) and chemotactic index (orientational accuracy).

### PBN Cytokine Release

Isolated PBNs were cultured for 18 h as previously described [23]. PBNs were stimulated with *F. nucleatum* (MOI 300:1), opsonised *S. aureus* (MOI 300:1) or with LPS (5 μg/ml per well) derived from *Escherichia coli* (Sigma, UK). Cells were pelleted by centrifugation (10 min, 2000 rcf) and supernatants were harvested and stored at – 80 °C for subsequent cytokine quantification. Pro-inflammatory cytokines interleukin-1β (IL-1β), interleukin-8 (CXCL8), interleukin-6 (IL-6) and tumour necrosis factor-α (TNFα) were quantified using Procartaplex™ multiplex immunoassays (Affymetrix eBioscence) from the first ten undiluted patient and control cell culture supernatants and also from patient plasma samples according to the manufacturer’s instructions. Mean fluorescence intensity (MFI) was measured on a Luminex 100/200 platform (USA) in the Clinical Immunology labs at the University of Birmingham. Plasma levels of the PBN serine protease myeloperoxidase (MPO) and the epithelial cell-derived neutrophil-activating peptide ENA-78 were measured by ELISA using commercially available kits (R&D systems, UK).

### Statistical Analysis

Weight loss was expressed as change in weight and excess % BMI loss between designated time points. All data was analysed statistically using GraphPad Prism 5 (version 5.0; GraphPad Software, CA, USA). Normality of data was determined using the Kolmogorov–Smirnov test, and all statistical comparisons were performed using non-parametric methods, as indicated in the text and figures. Previous studies of neutrophil functional assay report day-to-day variation [20, 24–26]. It was necessary to present the data as a ratio by dividing patient values by controls per result per time point.

## Results

### Patient Demographic

Twenty-three patients were enrolled onto the study and five chose to withdraw due to changes in surgical choice and for personal reasons. Eighteen patients (17 females, 1 male; mean age ± SD = 48 ± 11 years) underwent gastric band surgery. Initial mean referral weight and BMI of the patients were 129.5 kg ± 19.5 and 49 ± 7.3, respectively. At 3 months’ post-surgery, average weight was 119.7 ± 15.8 and excess % BMI loss was 14.6% ± 7. Demographic information is shown in Table 1 and Fig. 1 summarises patient withdrawal points.

**Table 1:** Characteristics of gastric band patients and controls

	Control	Patient
Female:male ratio	17:01	17:01
Mean age ± SD (range)	47 ± 9.8 (27–65)	48 ± 10.5 (24–67)
No. with a single morbidity	n/a	4
No. with > 2 co-morbidities	n/a	12
No. with diabetes (type 2)	n/a	7
No. taking lipid-lowering drugs	n/a	7
BMI at first blood collection (time point 1) mean ± SD (range)	23 ± 2.2 (19–27)	49 ± 7.3 (38–62)
BMI at second blood collection (time point 2) mean ± SD (range)	23 ± 2.2 (19–25.5)	47 ± 7.3 (36–59)
BMI at final blood collection (time point 3) mean ± SD (range)	23 ± 1.8 (20–26)	43 ± 6 (33–54)
Mean overall % weight loss (T1–3) ± SD (range)	n/a	14.6 ± 7.1 (0.17–30.2)

### Plasma Biochemistry

Comparisons between patients and controls demonstrated differences at T1 for HbA1c, HDL and LDL/HDL ratio (Table 2). Significance was evident between patient and controls for HbA1c (*p* < 0.001), HDL (*p* < 0.01) LDL/HDL (*p* < 0.05) and TG (*p* < 0.01) at time point 1. Significance was lost for all outcomes except HDL levels (*p* < 0.01) by T3, which remained significantly lower in patients versus controls.

**Table 2:** Blood biochemistry of patients and controls at each time point. Plasma was collected and the detected cytokines CXCL8 and TNFα were measured in patients (*n* = 10) and controls (*n* = 10) per time point respectively (*n* = 10). IL-1β and IL-6 were tested but not detected at high enough levels (data not shown). In addition, the neutrophil-activating peptide ENA-78 and enzyme MPO were also measured (*n* = 18). *n.d.* not detected. Data shown as mean, ±standard deviation and range. Statistical test: Wilcoxon matched-pairs. *p < 0.05, **p < 0.01, ***p < 0.001

Analyte	T1		T2		T3	
	Control	Patient	Control	Patient	Control	Patient
HbA1c	5.4 ± 1***	7.1 ± 1.4	5.1 ± 0.45***	6.7 ± 1.1	5.6 ± 0.77	5.5 ± 0.9
	(4.2–6.4)	(5.1–9.7)	(4.5–5.8)	(5.1–8.6)	(4.5–6.8)	(4.5–8.3)
Cholesterol	195 ± 43	166 ± 50	183 ± 43*	148 ± −47	183 ± 36	162 ± 43
	(136–264)	(114–336)	(110–257)	(100–305)	(132–279)	(113–256)
HDL	75 ± 15	54 ± 13**	68 ± 18	48 ± 14**	72 ± 16	58 ± 17*
	(48–100)	(37–84)	(35–100)	(33–84)	(41–100)	(36–84)
LDL	83 ± 37	84 ± 43	77 ± 37	80 ± 45	88 ± 36	82 ± 38
	(45–152)	(42–219)	(40–155)	(32–226)	(39–189)	(17–154)
LDL/HDL	1.2 ± 0.6	1.6 ± 0.8*	1.3 ± 0.8	1.8 ± 1.2*	1.3 ± 0.7	1.5 ± 0.7
	(0.5–2.3)	(0.5–3.8)	(0.5–3.3)	(0.4–5.3)	(0.5–2.8)	(0.2–2.7)
TG	95 ± 38	143 ± 62*	89 ± 49	121 ± −55*	91 ± 31	117 ± 56
	(50–187)	(63–292)	(50–244)	(51–262)	(50–170)	(50–250)
TNFα	12.5 ± 1.18	12.8 ± 3.49	n.d.	n.d.	n.d.	n.d.
	(10–14.3)	(11.5–15.7)				
CXCL8	3.27 ± 1.18	3.49 ± 2.35	1.78 ± 1.67	1.69 ± 1.67	1.39 ± 0.88	1.34 ± 0.78
	(2.4–5.3)	(1.59–9.2)	(0–5.2)	(0–5)	(0–2.54)	(0–2.39)
ENA-78	599.5 ± 516	1045.5 ± 1452.6	Not analysed	Not analysed	321.9 ± 262.2	66.6 ± 34.8***
	(121.1–1855.3)	(103.8–5234.1)			(66.2–998.1)	(34.7–169.6)
MPO	162.4 ± 66.3	199.7 ± 160.8	Not analysed	Not analysed	101.8 ± 60.9	18.6 ± 12.68***
	(30.3–241.7)	(28.1–652.5)			(29.6–303.8)	(8–49.6)

### NET Quantification

**Fig. 2:**
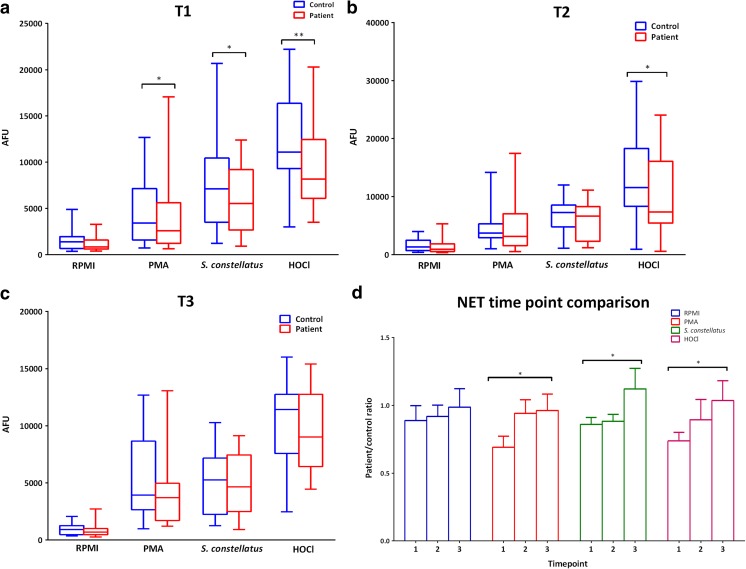
Patient and control NET release at different time points. PBNs were isolated and NETs were quantified in response to direct receptor-independent stimulation using PMA (50 nM) and HOCl (0.75 mM), and via the toll-like receptor (TLR) pathway *S. constellatus* (MOI 1 in 500). Data presented as box and whisker plots (a–c) for patient (red bars) and controls (blue bars) respectively. *p < 0.05, **p < 0.01. Statistical test for a–c was Wilcoxon matched-pairs. Data presented as patient/control ratios (d). *n* = 18 for total patient and controls respectively. Statistical test: Friedman and Dunn’s post-test

At T1 NET formation (Fig. 2(a)) was significantly lower in patient PBNs relative to controls for all stimuli employed (*p* < 0.05 and *p* < 0.01 for HOCl). Statistical significance remained at T2 (Fig. 2(b)) for HOCl (*p* < 0.05) and was lost completely by T3 (Fig. 2(c)). NET production was also compared between time points. There were significant differences in NET production by patient PBNs between T1 and T3 for PMA and HOCl, indicating that weight loss is associated with an increase in NET production. Overall NET production increased following weight loss in gastric band patients.

### ROS Generation

**Fig. 3:**
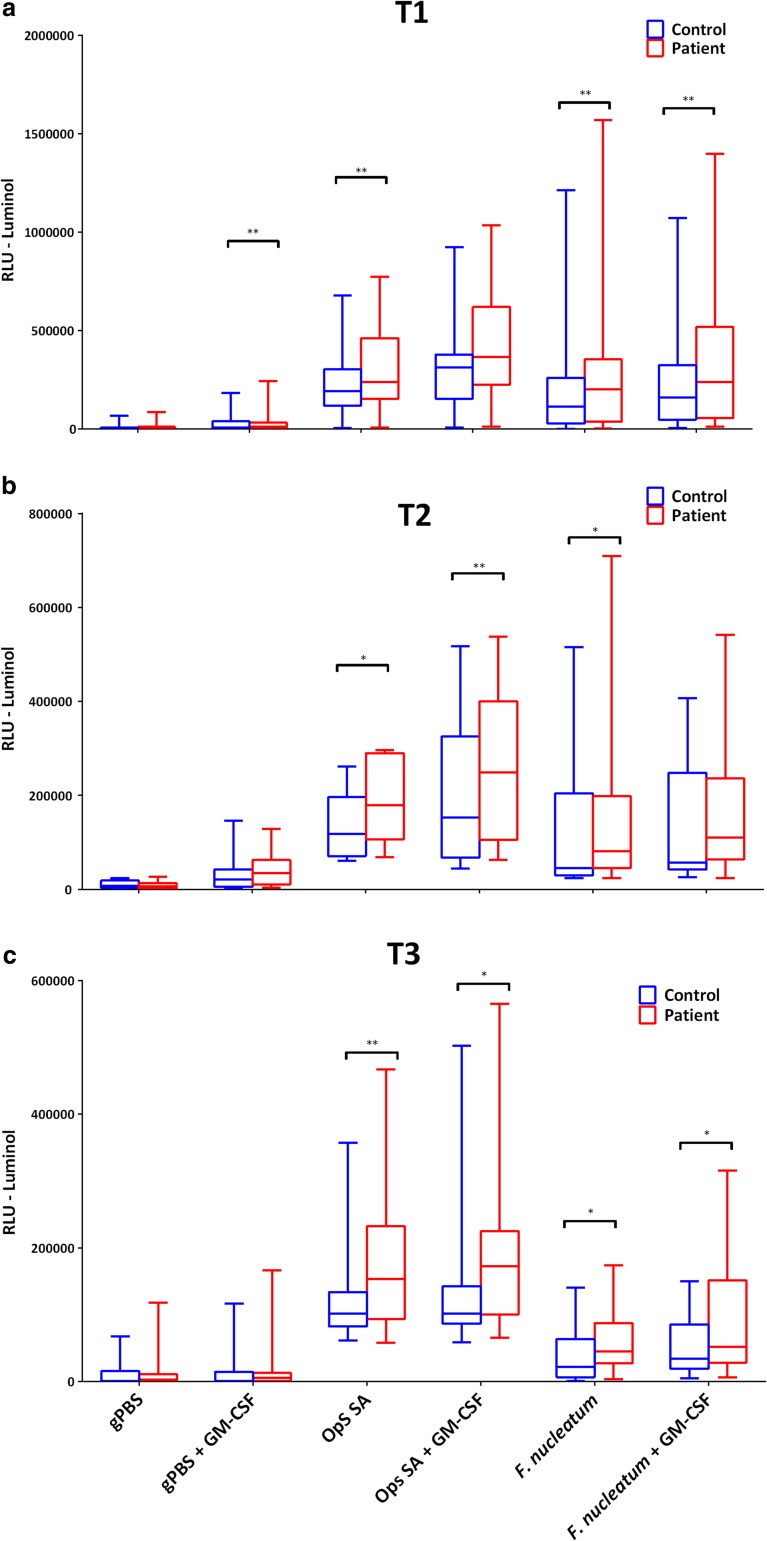
Patient and control ROS production at different time points. PBN ROS generation was detected following priming with/without GM-CSF (10 ng/ml) and stimulation using opsonised *S. aureus* (MOI 1 in 150) for Fcγ receptor stimulation and *F. nucleatum* (MOI 1 in 300) for TLR stimulation. Data presented as box and whisker plots. *n* = 18 for total patient (red bars) and controls (blue bars) respectively for time points 1 (a), 2 (b) and 3 (c). Statistical test was Wilcoxon matched-pairs. Data was also normalised (patient/control) and compared between the time points (d). Statistical test was Friedman and Dunn’s post-test, *p < 0.05, **p < 0.01

PBNs from gastric band surgery patients released significantly higher levels of ROS in the presence of a stimulus and following priming and stimulation at T1 with all three chemiluminescent reagents (Fig. 3). Over the course of the different study time points, significant differences remained for luminol (Fig. 3(a–c)) and were partially lost for isoluminol (Fig. 3(d–f)) and lucigenin (Fig. 3(g–i)). Overall, weight loss in the patients was accompanied by a reduction in the levels of ROS release when compared with healthy controls. There were no significant differences in ROS production by patient PBNs across the three time points (Supplementary Fig. 1).

### Chemotaxis

**Fig. 4:**
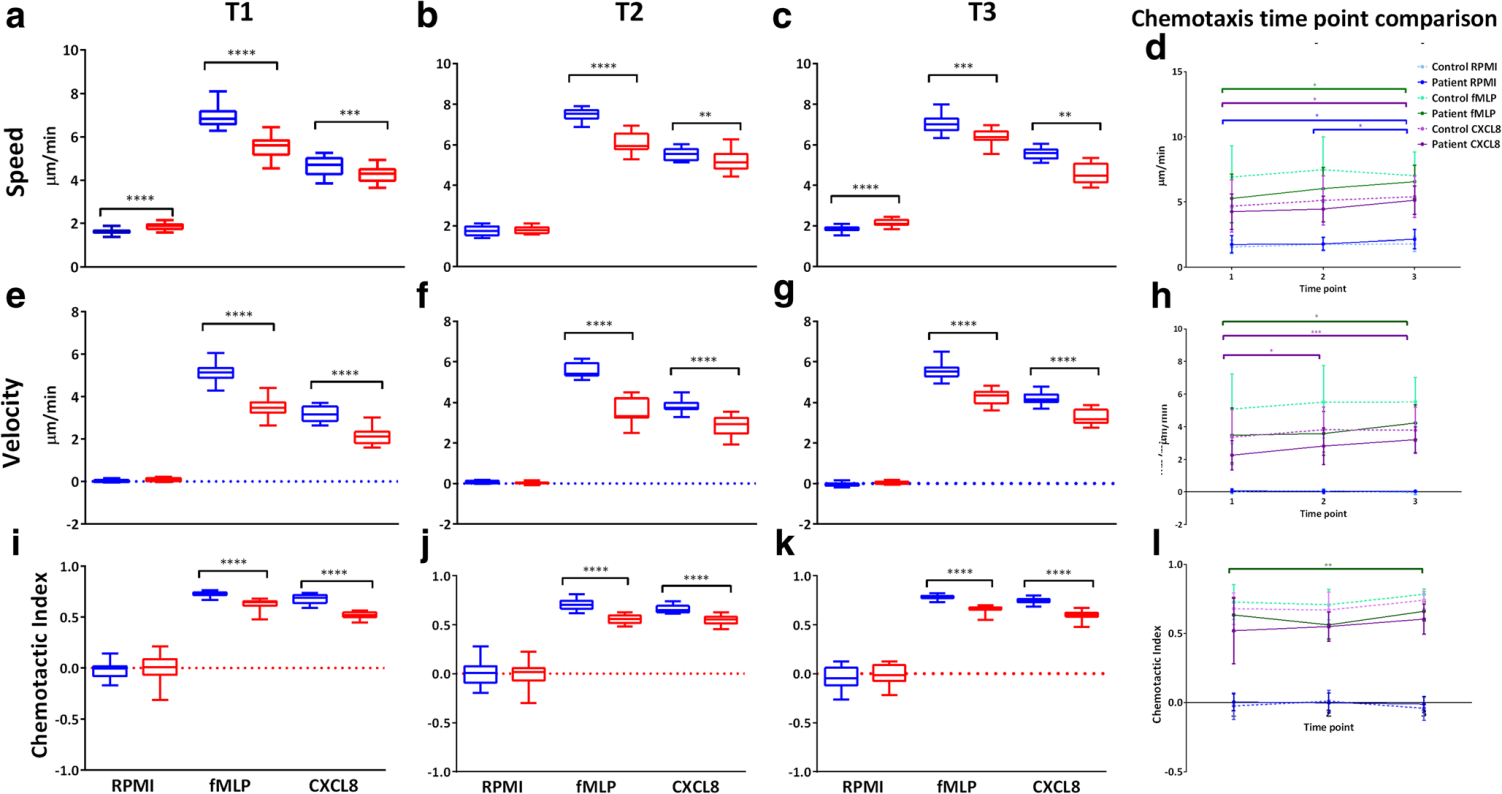
PBN chemotaxis in patients and controls at different time points. PBN chemotaxis was performed and cells analysed. Tracked information was used to generate average speed (a–c), velocity (e–g) and CI (i–k) for patient and control PBNs at each time point (1/2/3) in the presence of RPMI (negative control), fMLP (10 nM) and CXCL8 (200 ng/ml) chemoattractants. Data shown as box and whisker plots (*n* = 18; 15 cells tracked per chemoattractant/RPMI) for patient (red bars) and control (blue bars). Statistical test was Wilcoxon matched-pairs, *p < 0.05, ***p < 0.001, ****p < 0.0001. For time point comparison (d, h, l), the data is presented as raw values because of low numerical variability enabling statistical analyses without the need for data normalisation (as was done for the ROS and NET analysis). *n* = 18 for total patient and controls respectively. Statistical test was Friedman and Dunn’s post-test, *p < 0.05, **p < 0.01, ***p < 0.001

Significant differences were detected between patient and control PBNs at all the three time points for speed, velocity and CI, with patient PBN movement being lower compared to controls (Fig. 4). Weight loss in patients was not accompanied by improvements in PBN chemotactic accuracy relative to controls. Chemotaxis was also compared across the different time points in patients (Fig. 4(d, h, l)). There were significant differences between T1 and T3 with speed and velocity in response to both fMLP and CXCL8, with a trend towards an overall increase in speed from T1 to T3. There were no significant differences for control values across any of the time points. Weight loss following gastric band surgery was associated with an increase in chemotactic accuracy, but this did not reach control patient levels.

### Pro-inflammatory Cytokine Release

Pro-inflammatory cytokines IL-1β, IL-6, CXCL8 and TNFα were measured in both plasmas (Table 2) in addition to cell culture supernatants (Supplementary Fig. 2) representing potential localised and systemic effects for all the three time points respectively. Patient PBNs released significantly higher levels of cytokines, both in the absence and presence of stimuli at T1 compared with controls (*p <* 0.05) with some significant differences remaining at T2. There were no differences remaining between patients and controls by T3. The data indicate that weight loss is accompanied by a reduction in pro-inflammatory cytokine release.

Whilst there were no differences in the plasma concentrations of cytokines between patients and controls, the neutrophil-derived enzyme MPO and the neutrophil-activating peptide ENA-78 (Table 2) were significantly reduced (MPO, *p <* 0.0001, Wilcoxon matched-pairs; ENA-78, *p <* 0.001, Wilcoxon matched-pairs) following weight loss, suggesting an overall reduction in the systemic inflammatory burden.

## Discussion

The aim of this study was to comprehensively characterise for the first time potential changes in PBN behaviour following weight loss in morbidly obese individuals at three different time points: during the course of a VLCD, and pre- and post-gastric band surgery. As expected, overall BMI reduced following a VLCD and 3 months’ post-surgery (BMI loss was 4.5% at T2 and 14.6% at T3). However, despite the weight loss, many patients (*n* = 13) still had a BMI of > 40 at the end of the 3-month follow-up, which may lead to residual alterations in patients’ immune status and specifically, a residual low-grade chronic inflammatory state.

Lipid profiles of obese patients showed an improvement following weight loss when compared with controls within each time point. Improved lipid profiles have been reported in other studies of weight loss in obese patients [27–29], and whilst our results support this finding, the patient values are more subdued which could be a result of shorter follow-up monitoring relative to other studies which measure blood lipid levels for an extended period of time (up to 4 years). In addition, the small sample size in the present study, the limited information on diet practices following surgery and differences in blood collection times (morning versus afternoon) which can contribute to varied results [30] may underlie the subdued results presented here.

Patient NET release was significantly lower than non-obese controls prior to weight loss and NET production increased in patients relative to controls during the course of their weight loss. These results are in conflict with previous studies in which NET release was reported to be exaggerated during states of chronic inflammation, such as chronic obstructive airways disease [31] and certain vasculitides [32]. The underlying reasons for the results presented here could be due to the administration of anti-inflammatory medications which are commonly used as a treatment for obese patients [33] and have been demonstrated to lower NET formation [34].

Increased PBN ROS production has been reported to be associated with adiposity, leading to an elevation of systemic oxidative stress due to lipid peroxidation and direct oxidative injury [35, 36] affecting other organs including the liver and aorta [37]. Moreover, elevated lipid and glucose levels are reported to stimulate superoxide production via PKC-dependent activation of vascular NADPH oxidase in cultured endothelial cells [38]. Enhanced ROS release was detected in patient PBNs compared to control PBNs prior to weight loss in all the three chemiluminescent assays employed. Significant differences between patients and controls remained at both subsequent time points for total ROS release (luminol), indicating that despite the weight loss, PBNs from obese patients were still in a hyper-reactive state. Extracellular HOCl release (isoluminol) and superoxide (lucigenin) release reduced by T3 compared to controls, indicating a partial reduction in PBN hyper-reactivity following weight loss. This is consistent with previous studies investigating PBN ROS production in obese compared to lean individuals [39].

Few studies have investigated the effects of obesity on PBN chemotaxis during the course of weight loss. However, studies of chemotactic activity from type 2 diabetes patients report defective PBN functions including defective chemotaxis [40]. Advanced glycation end products (AGEs) have been shown to interfere with leukocyte function, inducing changes in intracellular calcium and actin polymerisation and subsequent impaired chemotaxis [41]. This suggests that exposure to AGEs, levels of which are known to be raised in obese individuals [42], may be a potential reason for the reduced PBN chemotactic accuracy detected in our study.

Pro-inflammatory cytokine release from PBN culture supernatants revealed significantly higher levels compared to controls with the stimuli used. Significance was evident with IL-1β, IL-6, CXCL8 and TNFα at T1 in the presence of various stimuli for TNFα only in the absence of a stimulus. These results are consistent with a hyperactive and hyper-reactive PBN phenotype, which was moderated to a degree by T3, when pro-inflammatory cytokine release in patients relative to control levels.

Longitudinal patient analysis revealed a reduction in IL-1β, IL-6 and TNFα over time for some stimuli, demonstrating that weight loss was associated with a decrease in systemic pro-inflammatory cytokine levels. These results are in agreement with reports within the literature regarding increased serum cytokine levels in obesity [43–45] reflecting a level of immune system activation. Systemic TNFα levels have been shown to correlate with increasing BMI [46]. TNFα has been shown to act directly upon adipocytes and altered levels have been suggested to interfere with glycaemic homeostasis and promote greater insulin resistance [47] as well as promoting endothelial dysfunction by inducing NF-κB signalling, NADPH activation and ROS production [48, 49]. Circulating pro-inflammatory cytokines have also been shown to prime PBNs [50] which may account for their enhanced production in our study when PBNs were stimulated. Other disorders of inflammation have also been reported to associate with enhanced PBN ROS release [51] in addition to enhanced pro-inflammatory cytokine production [52].

Several cell types have been shown to be exaggeratedly affected from the inflammatory insult during obese condition caused by nutrient overload, specifically increase circulating levels of fatty acids, on the metabolic cells. Effects on immune cell signalling include up-regulation of the genes encoding for cytokines, chemokines and other inflammatory mediators through activated transcription factors—nuclear factor-kB, activator protein-1, nuclear factor of activated T cells and signal transducer and activator of transcription 3 [53–55]. One recent study in mice fed a high-fat diet demonstrated reduced expression of peroxisome proliferator receptor gamma (PPARg), an anti-inflammatory nuclear factor, in bone marrow neutrophils; suppression of PPARg; increased neutrophil cytokine expression in blood neutrophils, and their infiltration into adipose tissues of high-fat diet-fed mice, in addition to increases p65/NFkB transcriptional activity [56]. To our knowledge, no studies have been conducted on PBN downstream signalling in obese individuals prior to and following gastric band surgery, and this represents an attractive avenue for further study.

MPO and ENA-78 were measured in plasma as markers of systemic or tissue neutrophil activation. ENA-78 is an epithelial cell-derived neutrophil-activating peptide that is increased in sites of inflammation. It acts as a chemoattractant for PBNs, has angiogenic properties and is reported to contribute to cancer progression [57]. The data presented here show a marked reduction in MPO and ENA-78 levels with weight loss, suggesting the overall positive effects of weight loss on the chronic inflammatory state that defines obesity.

Limitations of the reported studies include the relatively small sample size (*n* = 18 pairs of participants) and presence of co-morbidities within the obese patients; 11 patients suffered from obstructive sleep apnoea (OSA), a disorder characterised by repetitive pauses of breathing caused by partial or complete collapses of upper airways during sleep [58]. The associated potential for hypoxia may stimulate peripheral blood cells, including PBNs, and can lead to increased production of ROS [59], endothelial dysfunction [60] and pro-inflammatory cytokine production [61]. Due to limited patient background information, it was not possible to determine the OSA status of patients during the study or if the extent of weight loss had impacted on their condition. Another limitation is the relatively short post-operative follow-up time of 3 months owing to reduced number of clinical appointments patients have with the surgical team rendering blood collection impractical; future studies would be aimed at including additional donations for a more in-depth comparison of PBN functional change.

In summary, the reported studies have characterised PBN behaviour in obesity prior to and following interventional weight loss for the first time using novel assays of neutrophil function. The data reported supports the idea that circulating PBNs are involved in obesity-related inflammation and that normalisation of the PBN behaviour relative to healthy controls is reflected by improvements in overall health of the patients as measured in terms of reduced circulating pro-inflammatory cytokines and more favourable blood chemistry measurements. PBNs from obese patients exhibit higher ROS generation and release of cytokines, which appears to enhance the extent of local and systemic inflammation. Weight loss was associated with improved outcomes broadly characterised by lower ROS production and cytokine release relative to healthy controls. Chemotactic accuracy following weight loss did not improve suggesting either longer-term therapy or environmental factors are required to restore PBN-directed movement.

## Electronic Supplementary Material

Supplementary Figure 1 Patient and control ROS production compared between time points. ROS generation detected following priming with/without GM-CSF (10 ng/ml) and stimulation using opsonised *S. aureus* (MOI 1 in 150) and *F. nucleatum* (MOI 1 in 300 Data presented as box and whisker plots. *n* = 18. Data was also normalised (patient/control) and compared between the time points. Statistical test: Wilcoxon matched-pairs, * = (*p* < 0.05), ** = (*p* < 0.01). Statistical test: Friedman and Dunn’s post-test, * = (*p* < 0.05), ** = (*p* < 0.01). (GIF 109 kb)

High Resolution Image (TIFF 282 kb)

Supplementary Figure 2 Cytokine quantification from PBN cultures at different time points. PBNs were incubated with RPMI (negative control), opsonised *S. aureus* (FcγR stimulation pathway; MOI 1:150), *F. nucleatum* (TLR stimulation pathway; MOI 1 in 300) or LPS (TLR stimulation pathway). IL-1β (a/e/i), IL-6 (b/f/j), CXCL8 (c/g/k) and TNFα (d/h/l) were measured at each time point. Plasma was collected and the same cytokines were measured in patients and controls per time point respectively (levels that could not be detected were designated as 0). Blue hollow circles and filled red circles represent control (*n* = 10) and patient (*n* = 10) samples respectively. Statistical test: Wilcoxon matched-pairs, * = (*p* < 0.05), ** = (*p* < 0.01). Time points were compared as patient/control ratios (m/n/o/p) denoting the overall change in cytokine release between T1 and T3. Data is presented as patient/control ratios to account for day-to-day variability as was performed for the NETs/ROS analysis. Statistical test: Friedman and Dunn’s post-test, * = (*p* < 0.05). (GIF 101 kb)

High Resolution Image (TIFF 259 kb)
