# Supplementary figures and images for: Impact of Bariatric Surgical Intervention on Peripheral Blood Neutrophil (PBN) Function in Obesity
Source: Obes Surg. 2017 Dec 14;28(6):1611–21. doi: 10.1007/s11695-017-3063-1 (PMC5973997; doi:10.1007/s11695-017-3063-1)

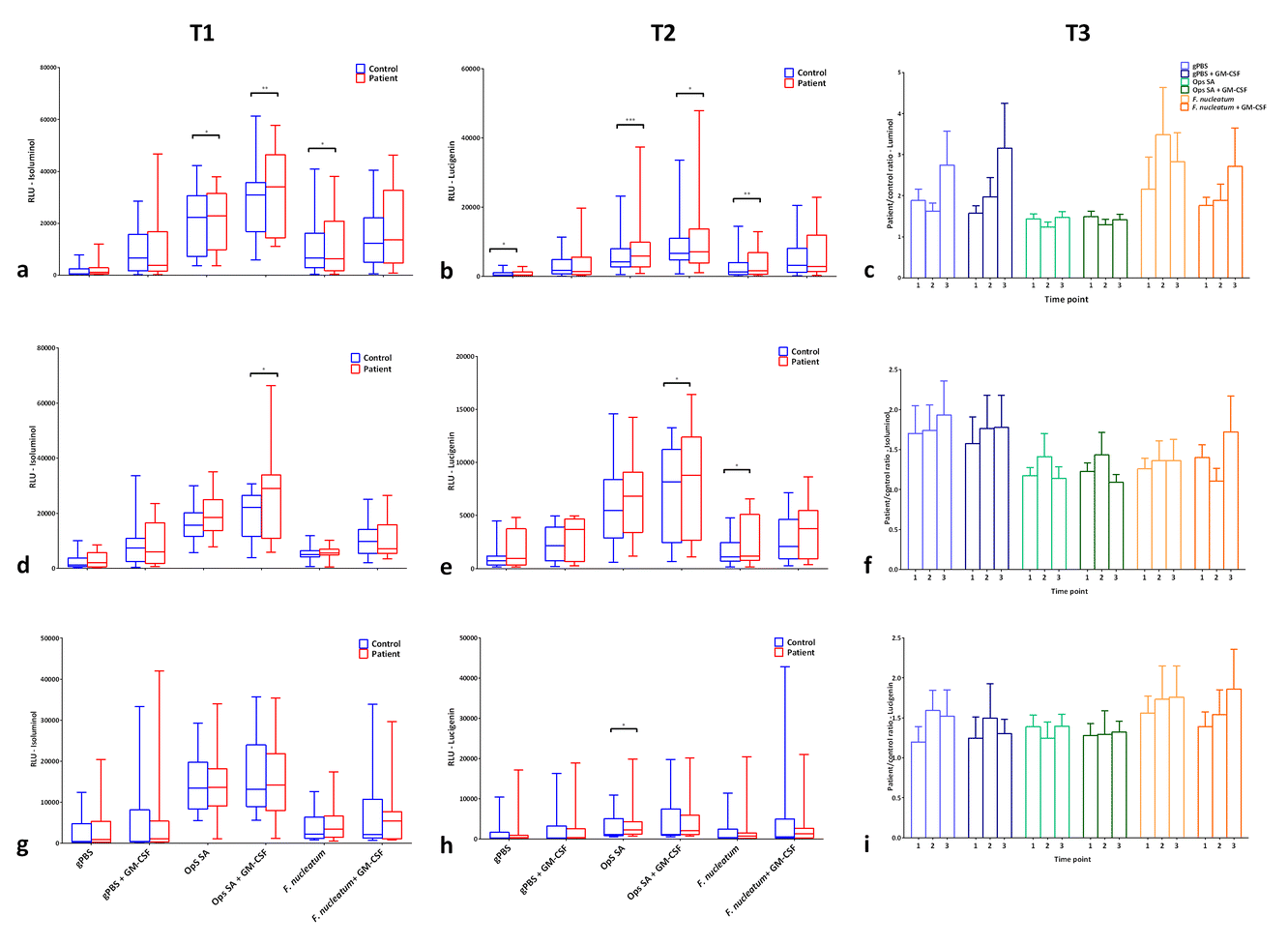

Supplement: Supplementary file 1 — Patient and control ROS production compared between time points. ROS generation detected following priming with/without GM-CSF (10 ng/ml) and stimulation using opsonised S. aureus (MOI 1 in 150) and F. nucleatum (MOI 1 in 300 Data presented as box and whisker plots. n = 18. Data was also normalised (patient/control) and compared between the time points. Statistical test: Wilcoxon matched-pairs, * = (p < 0.05), ** = (p < 0.01). Statistical test: Friedman and Dunn’s post-test, * = (p < 0.05), ** = (p < 0.01). (GIF 109 kb) [file 11695_2017_3063_Fig5_ESM.gif]

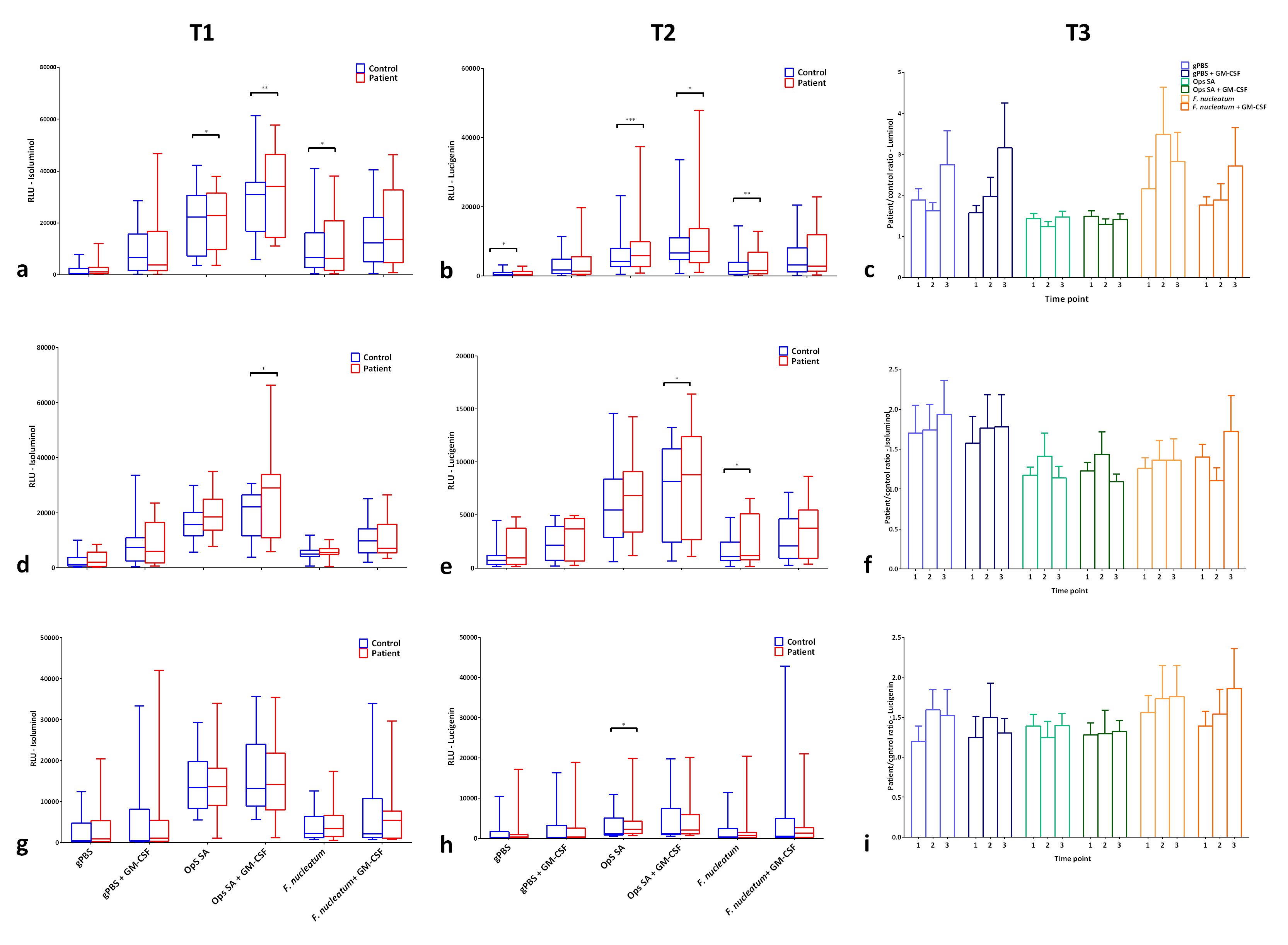

Supplement: Supplementary file 2 — (TIFF 282 kb) [file 11695_2017_3063_MOESM1_ESM.tif]

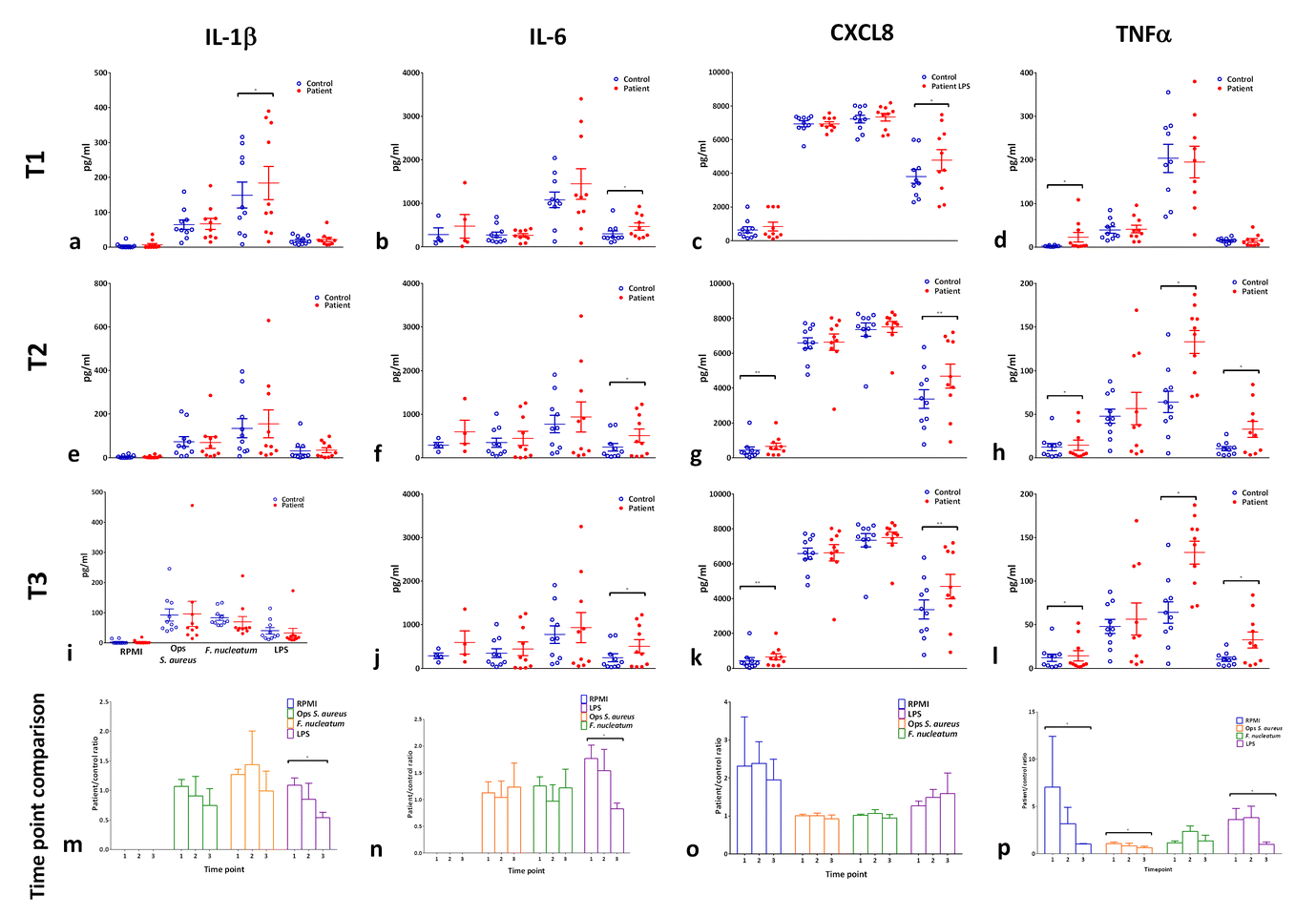

Supplement: Supplementary file 3 — Cytokine quantification from PBN cultures at different time points. PBNs were incubated with RPMI (negative control), opsonised S. aureus (FcγR stimulation pathway; MOI 1:150), F. nucleatum (TLR stimulation pathway; MOI 1 in 300) or LPS (TLR stimulation pathway). IL-1β (a/e/i), IL-6 (b/f/j), CXCL8 (c/g/k) and TNFα (d/h/l) were measured at each time point. Plasma was collected and the same cytokines were measured in patients and controls per time point respectively (levels that could not be detected were designated as 0). Blue hollow circles and filled red circles represent control (n = 10) and patient (n = 10) samples respectively. Statistical test: Wilcoxon matched-pairs, * = (p < 0.05), ** = (p < 0.01). Time points were compared as patient/control ratios (m/n/o/p) denoting the overall change in cytokine release between T1 and T3. Data is presented as patient/control ratios to account for day-to-day variability as was performed for the NETs/ROS analysis. Statistical test: Friedman and Dunn’s post-test, * = (p < 0.05). (GIF 101 kb) [file 11695_2017_3063_Fig6_ESM.gif]

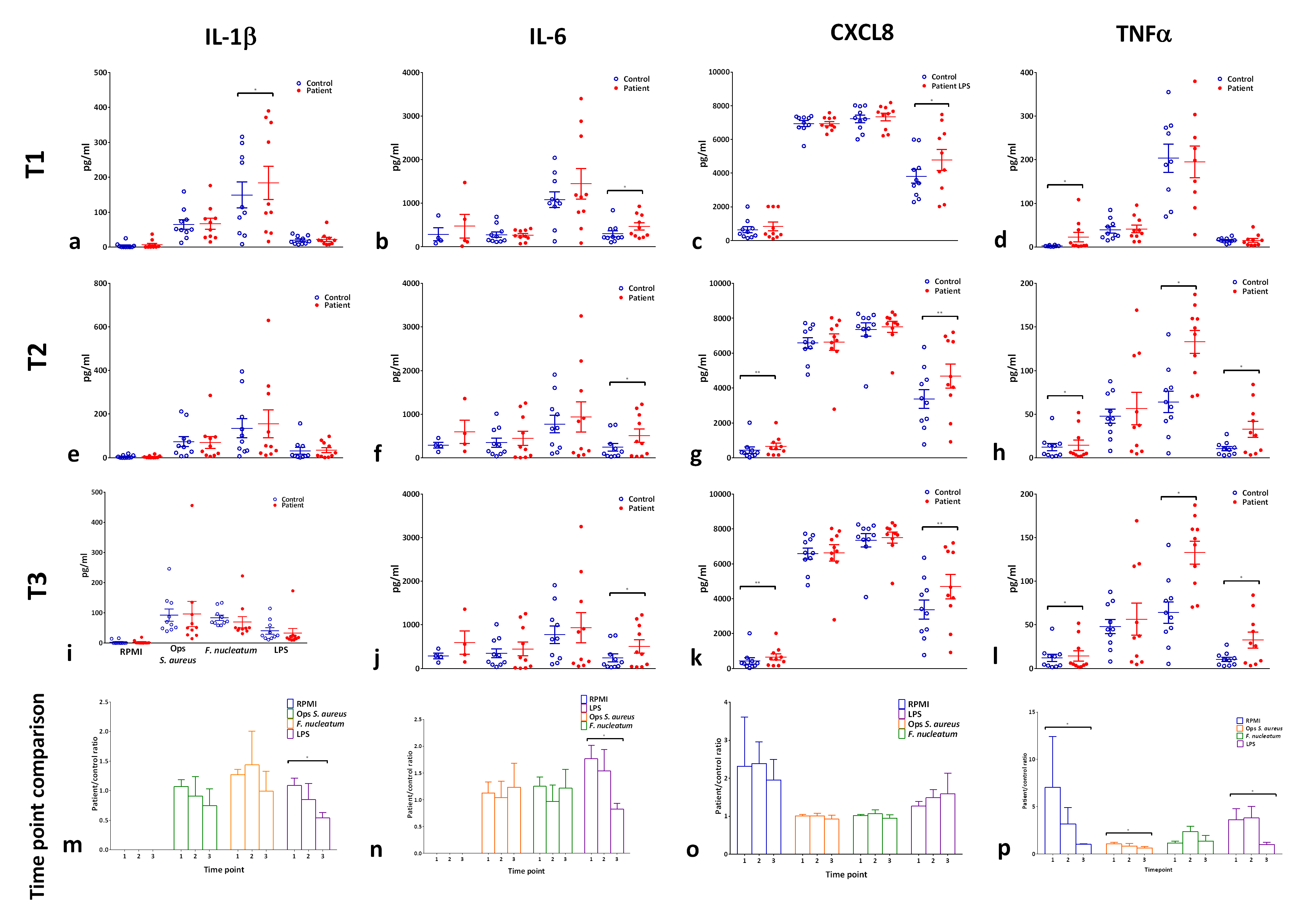

Supplement: Supplementary file 4 — (TIFF 259 kb) [file 11695_2017_3063_MOESM2_ESM.tif]
